# Timing and social patterning of weaning practices in Cyprus: BrEaST start in life longitudinal study

**DOI:** 10.1093/eurpub/ckac131.429

**Published:** 2022-10-25

**Authors:** M Economou, O Kolokotoni, E Hadjigeorgiou, I Paphiti-Demetriou, V Hadjiona, C Kouta, E Lambrinou, I Hadjipanteli, N Middleton

**Affiliations:** 1 Department of Nursing, Cyprus University of Technology, Limassol, Cyprus; 2 Cyprus Breastfeeding Association “Gift for Life”, Nicosia, Cyprus; 3 Archbishop Makarios Pediatric Hospital, Nicosia, Cyprus

## Abstract

**Background:**

Introduction of solids in the infant diet, according to WHO recommendations, should initiate onwards of sixth months. Evidence, however, suggests that solids introduction occurs earlier even if breastfeeding continues. With low breastfeeding rates, Cyprus is ranking last in Europe and little is known about the timing and socio-demographic pattern of solid introduction.

**Methods:**

Using the retrospective event calendar method at the 4th and 6th month after birth, the timing of initiation of solids was estimated among a consecutive sample of 350 mother-baby dyads from all public (N = 5) and 29 (of 35) private maternity clinics. The likelihood of early introduction according to socio-demographic characteristics and breastfeeding self-efficacy (measured at birth and 1st month) was estimated in logistic regression models.

**Results:**

Three out of ten women (30.8%) had initiated solids before the 4th month and only 20% hadn't by the 6th month. Almost half of non-Cypriot mothers (47.1%) initiated solids earlier than the 5th month, twice more likely compared to 25.1% of Cypriot mothers (OR: 2.45 95% CI: 1.30-4.57). There was a stepwise association with educational attainment with mothers with tertiary education more likely to initiate solids later (OR: 2.76 95% CI: 1.33-5.71) compared to those with at most secondary education. A similar social gradient was observed with income but was not statistically significant in multivariable models, while no association was observed with mode of birth (55.9% by C/S). Even though low breastfeeding self-efficacy at first month was predictive of earlier initiation, surprisingly, primiparous or multiparous mothers without previous breastfeeding experience were more likely to initiate solids later.

**Conclusions:**

Early introduction of solids with a clear social gradient suggests the need for strengthening the currently weak community-based interventions to improve weaning practices, including screening for breastfeeding self-efficacy.

**Key messages:**

• Four in five mums in Cyprus introduce solids before the 6th month with one in three introducing solids in the infant’s diet before the 4th month, with a clear social gradient in the patterning.

• Community-based intervention programmes are needed to improve weaning practices, including screening for low breastfeeding self-efficacy in a country with low breastfeeding rates.

